# Enhanced protein translocation to mammalian cells by expression of EtgA transglycosylase in a synthetic injector *E. coli* strain

**DOI:** 10.1186/s12934-022-01860-y

**Published:** 2022-07-02

**Authors:** Beatriz Álvarez, Víctor Muñoz-Abad, Alejandro Asensio-Calavia, Luis Ángel Fernández

**Affiliations:** 1 Department of Microbial Biotechnology, Centro Nacional de Biotecnología, Consejo Superior de Investigaciones Científicas (CNB-CSIC), Darwin 3, Campus Cantoblanco, 28049 Madrid, Spain; 2 Programa de Doctorado en Biociencias Moleculares, Universidad Autónoma de Madrid (UAM), Campus Cantoblanco, 28049 Madrid, Spain; 3 Present Address: Centro de Biología Molecular “Severo Ochoa” (Consejo Superior de Investigaciones Científicas – Universidad Autónoma de Madrid), Nicolas Cabrera 1, Campus Cantoblanco, 28049 Madrid, Spain

**Keywords:** *E. coli*, Type III secretion systems, Injectisomes, EtgA, SIEC, Peptidoglycan, Protein translocation

## Abstract

**Background:**

Bacterial type III secretion systems (T3SSs) assemble a multiprotein complex termed the injectisome, which acts as a molecular syringe for translocation of specific effector proteins into the cytoplasm of host cells. The use of injectisomes for delivery of therapeutic proteins into mammalian cells is attractive for biomedical applications. With that aim, we previously generated a non-pathogenic *Escherichia coli* strain, called Synthetic Injector *E. coli* (SIEC), which assembles functional injectisomes from enteropathogenic *E. coli* (EPEC). The assembly of injectisomes in EPEC is assisted by the lytic transglycosylase EtgA, which degrades the peptidoglycan layer. As SIEC lacks EtgA, we investigated whether expression of this transglycosylase enhances the protein translocation capacity of the engineered bacterium.

**Results:**

The *etgA* gene from EPEC was integrated into the SIEC chromosome under the control of the inducible *tac* promoter, generating the strain SIEC-eEtgA. The controlled expression of EtgA had no effect on the growth or viability of bacteria. Upon induction, injectisome assembly was ~ 30% greater in SIEC-eEtgA than in the parental strain, as determined by the level of T3SS translocon proteins, the hemolytic activity of the bacterial strain, and the impairment in flagellar motility. The functionality of SIEC-eEtgA injectisomes was evaluated in a derivative strain carrying a synthetic operon (eLEE5), which was capable of delivering Tir effector protein into the cytoplasm of HeLa cells triggering F-actin polymerization beneath the attached bacterium. Lastly, using β-lactamase as a reporter of T3SS-protein injection, we determined that the protein translocation capacity was ~ 65% higher in the SIEC-EtgA strain than in the parental SIEC strain.

**Conclusions:**

We demonstrate that EtgA enhances the assembly of functional injectisomes in a synthetic injector *E. coli* strain, enabling the translocation of greater amounts of proteins into the cytoplasm of mammalian cells. Accordingly, EtgA expression may boost the protein translocation of SIEC strains programmed as living biotherapeutics.

## Background

**Fig. 1:**
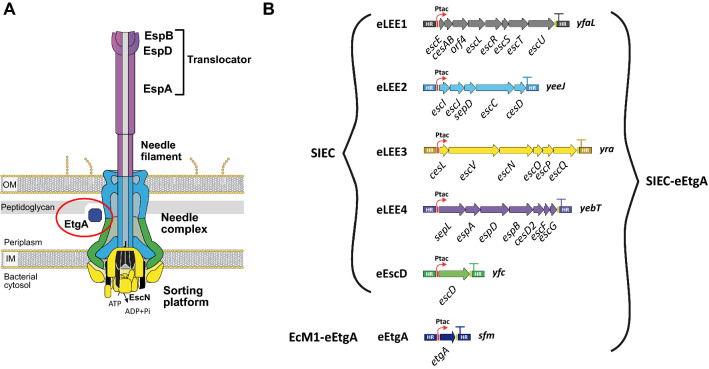
The T3SS injectisome of EPEC and the encoding operons in the engineered SIEC strains. **A** Scheme of the T3SS structure of EPEC. The lytic transglycosylase EtgA, which is associated with peptidoglycan, is marked with a red ellipse. **B** Synthetic transcriptional units of the T3SS under the control of the *tac* promoter (Ptac) in the strains SIEC, SIEC-eEtgA and EcM1-eEtgA. The chromosomal loci used as integration sites of the *eLEE* operons are indicated to the right of each transcriptional unit (described in [19] and in this study for eEtgA in the *sfm* locus). Different colors of injectisome components in **A** correspond to loci in **B**

Many Gram-negative bacterial pathogens, such as enteropathogenic *Escherichia coli* (EPEC) strains, use a type III secretion system (T3SS) to inject a specialized group of proteins known as effectors directly into the cytoplasm of host cells, which subvert multiple cellular functions and contribute to infection [1–3]. Protein effectors are translocated through a syringe-like multiprotein complex, the injectisome, which spans the bacterial envelope: the inner membrane (IM), the periplasm, the peptidoglycan, and the outer membrane (OM) (Fig. 1A) [4, 5]. The injectisome comprises a cytoplasmic sorting platform that is connected to a large proteinaceus multi-ring structure, the needle complex, crossing the bacterial envelope and projecting a needle filament towards the extracellular surface of the bacterium. A specialized ATPase (EscN in EPEC) is localized in the sorting platform of the injectisome and energizes protein secretion [6, 7]. Accordingly, null mutants in this ATPase do not assemble functional injectisomes [7, 8]. In EPEC strains, the needle complex is further expanded by a long extracellular protein filament of variable length (up to 700 nm) formed by multimerization of the EspA protein [9, 10]. The tip of the EspA filament is decorated by EspB and EspD proteins, which form a translocon pore in the membrane of the mammalian cell for passage of the effectors [11, 12]. EspA, EspB and EspD are collectively referred to as the translocators, and are actively secreted through the functional needle complex, being easily detected in the supernatants of EPEC cultures. All genes needed to assemble functional injectisomes in EPEC are encoded within a ~ 35-kb genetic locus called the locus of enterocyte effacement (*LEE*) [2, 13].

The assembly of the injectisome in EPEC is assisted by the lytic transglycosylase EtgA, a periplasmic enzyme encoded in the *LEE* that degrades the peptidoglycan and facilitates the insertion of the multiprotein complex into the bacterial envelope [14]. EtgA cleaves the β-1,4 glycosylic bond between the N-acetylglucosamine and N-acetylmuramic acid residues in the peptidoglycan [15]. While the activity of EtgA contributes to the assembly of functional injectisomes, it is not essential in EPEC. Deletion of the *etgA* gene in this pathogen causes a ~ 40% reduction in assembled injectisomes but not a total loss, likely due to functional redundancy of EtgA activity with other lytic transglycosylases expressed by this bacterium [14].

Owing to their protein translocation capabilites, T3SSs have great potential in biomedical applications for the delivery of therapeutic proteins into mammalian cells [16–18]. Several studies have demonstrated the utility of T3SS for translocation of protein antigens, single-domain antibodies, cellular transcription factors, or apoptotic protein domains (for review, see [17]). Despite these promising results, however, the translation of a T3SS-based protein delivery platform into the clinical setting might be hampered by the fact that only pathogenic bacteria produce this secretion system. In this case, the use of attenuated strains is not a solution, as the biosafety of future therapies could be compromised. To overcome this limitation, we previously generated a genetically-modified non-pathogenic *E. coli* strain that efficiently assembles filamentous injectisomes from EPEC [19]. This strain, named Synthetic Injector *E. coli* (SIEC), was constructed from the commensal *E. coli* K-12 strain EcM1, a derivative of MG1655 [20] with a deletion in the type I fimbriae operon (*fim*) [21, 22]. To obtain SIEC, five engineered operons (eLEE1 to eLEE4 and eEscD) were constructed containing all *LEE* genes encoding the structural proteins of the injectisome as well as protein components needed for its function (e.g., chaperones and translocators), but not the effectors or transcriptional regulators of the T3SS found in the *LEE* [23, 24]. The engineered operons containing a strong ribosome-binding sequence (RBS) for translation initiation [25] were placed under the control of the isopropyl β-d-1-thiogalactopyranoside (IPTG)-inducible *tac* promoter (Ptac) [26] and were integrated into five different *loci* of the EcM1 chromosome (Fig. 1B) [19]. SIEC has been proven to efficiently assemble functional injectisomes similar to those of EPEC and to translocate the effector protein Tir (translocated intimin receptor) into mammalian cells [19]. It was designed with the minimal number of genes needed for the assembly of functional injectisomes. Thus, the auxiliary gene *etgA* was not included in the SIEC strain, even though it was also localized in the *LEE*. In agreement with the non-essentiality of EtgA for injectisome assembly in EPEC [14], SIEC formed functional injectisomes without the lytic transglycosylase activity of EtgA [19].

In this study, we aimed to increase the translocation efficiency of SIEC by expressing EtgA. To achieve this, we generated a new strain, SIEC-eEtgA, which has the *etgA* gene integrated into the SIEC chromosome under the control of the *tac* promoter. Our results establish that SIEC-eEtgA produces greater amounts of assembled injectisomes than the parental SIEC strain, which leads to enhanced protein translocation efficiency into the cytoplasm of mammalian cells. SIEC-eEtgA represents an improved bacterial strain for protein delivery into mammalian cells by engineered live biotherapeutics [27].

## Results and discussion

### Generation of the strains SIEC-eEtgA and EcM1-eEtgA

It has been reported that forced overexpression of the transglycosylase EtgA severely affects the viability of *E. coli* owing to its uncontrolled lytic activity on peptidoglycan [15]. To minimize potential deleterious effects, we placed the *etgA* gene under the control of the IPTG-inducible Ptac promoter and the endogenous RBS of *etgA*, generating the transcriptional unit eEtgA. This genetic construct was integrated into the chromosome of SIEC replacing the *sfm* locus, generating the strain SIEC-eEtgA (Fig. 1B). The *sfm* locus encodes a cryptic chaperone-usher fimbrial adhesin in *E. coli* K-12 [28], and insertion of a Ptac-*gfp* reporter construct in this locus produced low expression levels of green fluorescent protein (GFP) [19]. The transcriptional unit eEtgA was also integrated at the *sfm* locus of the EcM1 chromosome, the parental strain of SIEC, generating the control strain EcM1-eEtgA and allowing us to study the effects of EtgA in absence of functional injectisomes.

### EtgA expression enhances injectisome assembly in SIEC-eEtgA

**Fig. 2:**
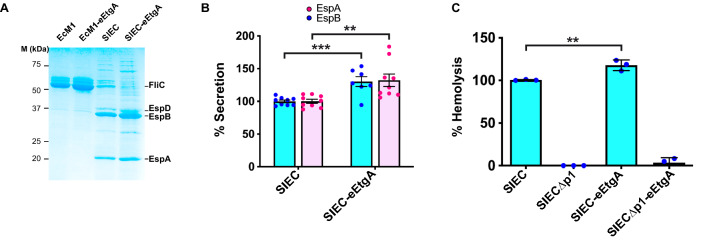
Secretion of translocator proteins EspA, EspB, EspD and hemolytic activity by SIEC-eEtgA. **A** Coomassie-stained SDS-PAGE of proteins found in culture supernatans of the indicated strains (EcM1, EcM1-eEtgA, SIEC, and SIEC-eEtgA). Bands corresponding to the proteins FliC, EspD, EspB and EspA are labeled. Mass (in kDa) of protein standards is indicated to the left. **B** Relative quantification of secreted EspA and EspB translocator proteins in SIEC-eEtgA relative to their levels in SIEC (considered 100%). Histogram shows the means and standard errors of the relative percentage of the secretion of each protein. Data were obtained from nine independent experiments. **C** Histogram showing the relative hemolytic activity of the indicated strains (SIEC, SIECΔp1, SIEC-eEtgA, and SIECΔp1-eEtgA). The hemolysis obtained with a control strain (EcM1) was subtracted from the represented values. SIEC hemolysis values were taken as 100%. Data represent the means and standard errors of three independent experiments. Statistical analysis was performed using the two-tailed Student’s *t* test. Asterisks indicate p-value < 0.01 (**) and < 0.001 (***)

The secretion into the culture medium of the filament protein EspA and the translocon proteins EspB and EspD indicates the correct assembly of functional injectisomes [19]. Analysis of concentrated supernatants of induced cultures by Coomassie-stained SDS-PAGE revealed that SIEC-eEtgA secreted these proteins, demonstrating the assembly of injectisomes in this strain (Fig. 2A). Quantification of the amounts of secreted EspA and EspB proteins revealed that the SIEC-eEtgA strain secreted ~ 30% higher levels than the SIEC strain (Fig. 2B). This suggests that EtgA can enhance the amount of assembled injectisomes in SIEC to a level similar to that reported for EtgA in EPEC [14].

We further evaluated the enhanced assembly of injectisomes in SIEC-eEtgA by comparing its hemolytic capacity with that of the parental SIEC strain. Injectisome translocon proteins form pores on erythrocyte membranes, which trigger hemolysis [14, 19, 29]. We performed quantitative hemolysis assays using the SIEC and SIEC-eEtgA strains and the isogenic negative control strains (SIECΔp1 and SIECΔp1-eEtgA) lacking the promoter of the *eLEE1* operon, which is essential for the production of injectisomes [19]. SIECΔp1-eEtgA was obtained by inserting the eEtgA into the *sfm* locus of SIECΔp1. Both negative control strains failed to show any detectable hemolytic activity (Fig. 2C). When we compared SIEC and SIEC-eEtgA strain, the latter showed a higher hemolytic activity (Fig. 2C), in agreement with the higher level of secreted EspA and EspB proteins and the hemolytic activities reported in wild-type and Δ*etgA* strains of EPEC [14]. Taken together, the results demonstrate that SIEC-eEtgA secretes higher amounts of the T3SS translocon proteins than the parental SIEC strain, suggesting the enhanced assembly of SIEC injectisomes by EtgA expression, similar to its reported activity in EPEC [14].

### EtgA expression decreases the motility but not the viability of SIEC-eEtgA strain

**Fig. 3:**
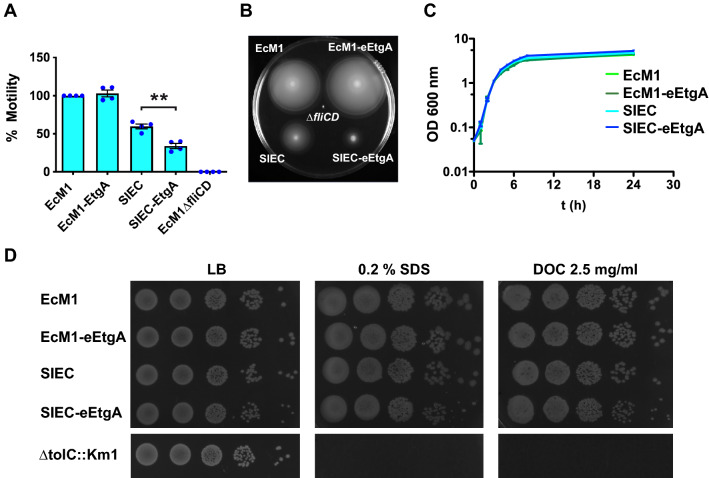
Motility and viability of EcM1-eEtgA and SIEC-eEtgA. **A** Quantification of the motility of different *E. coli* strains in LB-soft agar plates containing the inductor IPTG (0.1 mM). The histogram represents the relative percentages of motility using the motility of the control strain EcM1 as 100%. The non-motile strain EcM1Δ*fliCD* was used as a negative control. The means and standard errors from four independent experiments are shown. Statistical analysis was performed using the two-tailed Student’s *t* test. Asterisks (**) indicates p-value < 0.01. **B** Image of a representative motility assay on LB-soft agar with IPTG. **C** Growth curve of the indicated *E. coli* strains (EcM1, EcM1-eEtgA, SIEC, and SIEC-eEtgA) on LB supplemented with IPTG. **D** Viability test (serial tenfold dilutions to determine colony forming units of the cultures) of the indicated strains (left) on LB agar plates supplemented with IPTG or additionally containing 0.2% (w/v) SDS or 2.5 mg/ml deoxycholate (DOC), as indicated. An *E. coli* K-12 Δ*tolC*::Km1 strain susceptible to SDS and DOC was used as a control

We previously reported that the expression of injectisomes in SIEC reduces the secretion of the major flagellar component, flagellin FliC, and suppresses bacterial motility [19]. This is likely caused by the interference of the components of the *eLEE1* operon [19], encoding basal components of T3SS, with similar components of the flagellar basal apparatus [1, 30]. Consistent with our previous data, the motility of SIEC was reduced by ~ 40% compared with the EcM1 parental strain (Fig. 3A, B). As could be expected by the increased injectisome expression, EtgA further reduced flagellar motility in SIEC, with a decrease of ~ 66% when compared with EcM1 (Fig. 3A, B). Notably, the motility of EcM1 was unchanged by the expression of EtgA (Fig. 3A, B, EcM1-eEtgA), indicating that the suppression of flagellar activity in SIEC-eEtgA is due to the greater expression of T3SS components and not to EtgA per se. The evident changes in bacterial motility is in good agreement with the levels of FliC secreted by these strains. Whereas EcM1-eEtgA and EcM1 strains showed similar high levels of flagellin, the levels of secreted FliC were much lower in SIEC and were further reduced in SIEC-eEtgA (Fig. 2A).

We also found that the growth curves of the strains EcM1, EcM1-eEtgA, SIEC and SIEC-eEtgA were superimposable (Fig. 3C), ruling out the possibility that the changes in motility observed in SIEC and SIEC-eEtgA were due to a growth defect. This result also demonstrates that EtgA expression from a single-copy gene in the chromosome of EcM1 or SIEC does not impact the growth of these strains. In line with this, the viability (CFU/ml) of SIEC and SIEC-eEtgA were similar, as determined by plating serial dilutions of induced cultures on LB agar plates (Fig. 3D). Further, EtgA-expressing and parental strains showed a similar viability in the presence of detergents (SDS) and bile salts (deoxycholate, DOC), indicating that the integrity of the bacterial envelope is maintained upon expression of EtgA (Fig. 3D). As expected, an *E. coli* K-12 strain with a deletion in *tolC* (Δ*tolC::km1*) was sensitive to the presence of SDS or DOC in the growth medium (Fig. 3D) [31].

In contrast to our results, the overexpression of EtgA in the BL21(DE3) strain of *E. coli* was found highly toxic and triggered cell lysis [15]. This difference is likely explained by the lower expression of EtgA in our strain, from a single-copy *etgA* integrated at the *sfm* locus of the *E. coli* K-12 chromosome, and not from a multicopy plasmid with the strong T7 promoter in the BL21(DE3) strain [15]. In addition, it has been reported that EtgA interacts with EscI [15], a protein that polymerizes in the inner rod of the injectisome, creating a channel across the cell wall (see Fig. 1A). This interaction restrains the activity of EtgA in a spatial manner where the injectisome is assembled. Thus, co-expression of EscI and EtgA in SIEC-eEtgA may localize the activity of EtgA at injectisome assembly sites in the cell wall. Additionally, EtgA activity is significantly reduced in the absence of EscI [15], suggesting that the peptidoglycan lysis occurs only under conditions of overexpression. Supporting this hypothesis, we found that growth, viability, and cell envelope integrity of the EcM1-eEtgA strain, which lacks EscI but expresses EtgA from the chromosome of *E. coli*, were also unaffected upon induction of EtgA (Fig. 3).

### Translocation of Tir effector into mammalian cells by SIEC expressing EtgA

**Fig. 4:**
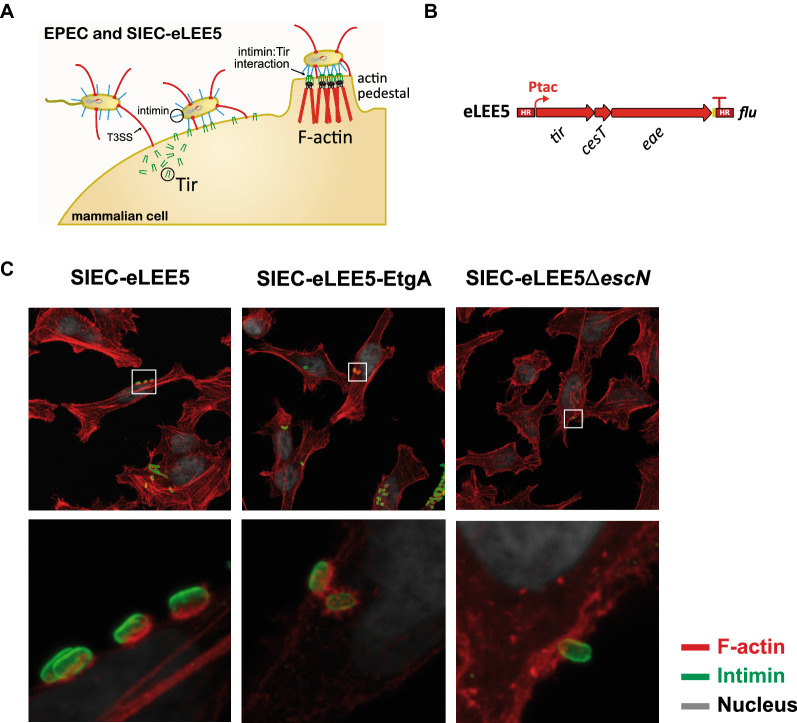
Translocation of Tir into mammalian cells by SIEC-eLEE5-eEtgA. **A** Graphic representation of the Tir translocation process. EPEC and SIEC-eLEE5 can inject Tir into the cytoplasm of mammalian cells through injectisomes. Tir is then exposed on the surface of the cell membrane where it interacts with intimin of the bacteria, leading to the polymerization of F-actin filaments and the formation of pedestal-like structures beneath the bacterium. **B** Scheme of the synthetic operon *eLEE5* containing the coding genes for Tir (*tir*), the chaperone CesT (*cesT*) and intimin (*eae*) under the control of the tac promoter (Ptac). This operon is integrated at the *flu* site of the chromosome. **C** Representative confocal fluorescence microscopy images of HeLa cells infected with the indicated bacterial strains (SIEC-eLEE5, SIEC-eLEE5-eEtgA and SIEC-eLEE5Δ*escN*). F-actin is stained with TRITC-conjugated phalloidin (red), and DNA and nuclei are stained with DAPI (grey). Bacteria are labeled with rabbit polyclonal anti-intimin280 and ALEXA488-conjugated anti-rabbit antibodies (green). The F-actin accumulations can be visualized as strong red fluorescence signals associated with attached bacteria, indicating the translocation of Tir protein. Bottom images are magnifications of the regions in the upper images marked with white squares

During the infection process, EPEC injects more than 24 effectors into enterocytes to subvert cell functions. One of these effectors is the translocated intimin receptor (Tir) [3, 32, 33], which localizes to the host cell plasma membrane, exposing a small domain (TirM) to the cell surface that acts as a receptor for the EPEC outer membrane protein intimin [34, 35] (Fig. 4A). The intimin-Tir interaction induces bacterial attachment and triggers the polymerization of F-actin on the host cell cytosol, generating actin-rich pedestal-like structures beneath the attached bacteria [36]. Actin pedestals can be easily visualized by fluorescence microscopy staining with a phalloidin-fluorophore conjugate. We previously reported actin pedestal formation in HeLa cells infected with SIEC bacteria carrying the synthetic operon *eLEE5*, which encodes intimin, Tir and its chaperone CesT (Fig. 4B) [19]. To confirm the protein translocation of SIEC bacteria expressing eEtgA to mammalian cells, we constructed a derivative of SIEC-eLEE5 with eEtgA inserted into the *sfm* locus. We found that, similar to SIEC-eLEE5, the SIEC-eLEE5-eEtgA strain triggered the formation of actin pedestals in HeLa cells (Fig. 4C). As expected, a negative control strain SIEC-eLEE5Δ*escN*, which lacks the ATPase EscN required for functional injectisomes, failed to induce actin pedestals in HeLa cells (Fig. 4 C). These data demonstrate that EtgA expression does not interfere with the protein translocation capacity of SIEC.

### EtgA enhances protein translocation into mammalian cells

**Fig. 5:**
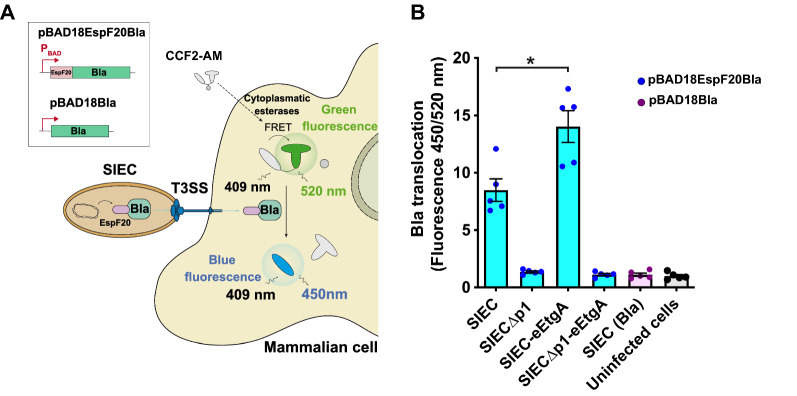
β-Lactamase translocation is enhanced in SIEC-eEtgA. **A** Scheme of β-lactamase (Bla) translocation by the SIEC strain fused to the T3S-signal EspF20. The CCF2 substrate is cleaved by the β-lactamase inside the mammalian cell, switching the emission of green (520 nm) fluorescence to blue (450 nm) fluorescence. On top left, the constructs EspF20-Bla and Bla (lacking T3S-signal) are represented in the pBAD18-derivatives under the l-arabinose-dependent P_BAD_ promoter [43]. **B** Histogram showing the Bla translocation assay results with different SIEC-derived strains expressing Esp20-Bla, or SIEC (Bla) control without the EspF20 signal. The strains SIECΔp1 and SIECΔp1-eEtgA do not assemble functional injectisomes and are used as negative controls. The results are the means and standard errors of five independent experiments. Statistical analysis was performed using the two-tailed Student’s *t* test. The asterisk indicates p-value < 0.05

Given that EtgA increases the assembly of injectisomes in SIEC, we next investigated whether it also enhances protein translocation into mammalian cells. To do this, we performed a quantitative protein translocation assay based on the activity of translocated β-lactamase (Bla) in the cytosol of mammalian cells (Fig. 5A) [37], which hydrolyzes the fluorescence resonance energy transfer (FRET) substrate CCF2-AM (a hydroxycoumarin acceptor linked to a fluorescein donor molecule by a cephalosporin bond). CCF2-AM diffuses into mammalian cells where cytoplasmic esterases release the negatively charged CCF2 moiety, which accumulates in the cytosol. Translocation of Bla into the cytosol of the mammalian cell leads to cleavage of CCF2, switching its fluorescence emission from green (520 nm) to blue (450 nm) upon excitation with a 409 nm wavelength laser. Accordingly, the ratio of fluorescence emission at 450 vs. 520 nm quantifies the levels of translocated Bla. To enable Bla translocation, its mature sequence (devoid of its endogenous N-terminal signal peptide) [38] was fused to the first N-terminal 20 amino acids of the EspF effector (EspF20). This N-terminal T3-signal has been used previously to translocate Bla and single-domain antibodies through the injectisomes in EPEC [37, 39]. Both the EspF20-Bla fusion and the Bla polypeptide lacking the EspF20 signal were expressed in SIEC from l-arabinose (ARA)-inducible promoters in plasmids pBAD18EspF20-Bla and pBAD18Bla, respectively (Fig. 5 A). SIEC-eEtgA and its isogenic SIEC parental strain (as a control) were transformed with these plasmids, induced with IPTG and ARA, and then used to infect cultured HeLa cells. The activity of translocated Bla in infected HeLa cells was quantified (see Materials and Methods), revealing that the SIEC and SIEC-eEtgA strains with functional injectisomes were capable of injecting EspF20-Bla into HeLa cells in a T3SS-dependent manner, as the control protein Bla without the T3-signal was not translocated (Fig. 5B). In addition, the T3S-negative control strains SIECΔp1 and SIECΔp1-eEtgA were unable to translocate EspF20-Bla. Notably, these experiments revealed that SIEC-eEtgA injected ~ 65% more EspF20-Bla than its parental SIEC strain (Fig. 5B), demonstrating that EtgA not only enhances the assembly of injectisomes in the bacterium, but also the level of protein translocation to mammalian cells.

## Conclusions

SIEC is a non-pathogenic *E. coli* strain expressing the filamentous injectisomes of EPEC for delivery of protein payloads into the cytoplasm of mammalian cells [19]. Here, we demonstrate that expressing the lytic transglycosylase EtgA from EPEC enhances the protein translocation capacity of SIEC. EtgA is a periplasmic protein encoded in the *LEE* of EPEC that has been reported to increase the assembly of injectisomes in EPEC [14]. We show that EtgA also aids in the assembly of EPEC injectisomes in SIEC without affecting its growth, viability, or integrity of its cell envelope. Analogous to the reported contribution of EtgA for the assembly of injectisomes in EPEC [14], the presence of EtgA in SIEC-eEtgA significantly increases the amount of assembled injectisomes (~ 30%) as determined by the quantification of secreted EspA and EspB proteins and the hemolytic activity on erythrocytes triggered by these pore-forming translocators. In addition, the expression of EtgA in SIEC further reduced the flagellar motility of the bacterium in comparison with the parental strain EcM1 (from ~ 40% reduction in SIEC to ~ 66% reduction in SIEC-eEtgA). This is likely caused by the interference of the components of the eLEE1 operon [19] with components of the flagellar basal body [1, 40]. Our study also establishes the functionality of the injectisomes assembled in SIEC-eEtgA. We demonstrate the translocation of the Tir effector into mammalian cells using a derivative of SIEC-eLEE5 [19] carrying eEtgA. Tir delivery triggered F-actin polymerization in the cytoplasm of HeLa cells beneath the attached bacterium. Quantification of the protein translocation levels in SIEC-eEtgA using a Bla reporter fused to the first 20 amino acids of the EspF effector (EspF20) as a T3S-signal [37, 39] revealed that SIEC-eEtgA translocated ~ 65% more EspF20-Bla fusion into HeLa cells than its parental SIEC strain, in agreement with the higher level of assembled injectisomes and demonstrating the enhanced capacity of SIEC-eEtgA to inject proteins into the cytoplasm of mammalian cells. Hence, SIEC-eEtgA represents an optimized bacterial platform for the efficient translocation of proteins into mammalian cells in future therapeutic strategies [27].

## Methods

### Bacterial strains and culture conditions

The strains and plasmids used in this study are listed in Table 1. The *E. coli* strains DH10BT1R and BW25141 were used for propagation and genetic manipulation of pBAD and *pir*-dependent pGE plasmids, respectively. Unless otherwise indicated, all the *E. coli* strains were cultured in Lysogenic Broth (LB) at 37 °C with shaking at 250 rpm. LB with 1.6% (w/v) agar was used for culturing on solid medium. When indicated, the culture medium was supplemented with IPTG, 0.4% (w/v) ARA, and appropriate antibiotics (50 µg/ml kanamycin [Km] and 30 µg/ml chloramphenicol [Cm]). Antibiotics were obtained from Duchefa-Biochemie.

**Table 1:** Bacterial strains and plasmids used in this study

Name	Genotype and description	Source or references
*E. coli* strains		
DH10B-T1R	(F^−^ λ^−^) *mcrA* Δ*mrr-hsdRMS-mcrBC φ80lacZDM15* Δ*lacX74 recA1 endA1 araD139* Δ(*ara, leu*)7697 *galU galK rpsL* (StrR) *nupG tonA*	Novagen
BW25141	(F- λ-) Δ*(araD-araB)567*, Δ*lacZ4787*(::rrnB-3), Δ*(phoB-phoR)580*, *galU95*, Δ*uidA3*::*pir*, *recA1*, *endA9*(del-ins)::*FRT*, *rph-1*, Δ*(rhaD-rhaB)568*, *hsdR51*	[46]
UT5600	K-12 (F^−^ λ^−^) Δ*(ompT-fepC)266*	[45]
UT5600ΔtolC::Km1	UT5600 Δ*tolC::Km1*	This study
EPEC	EPEC O127:H6 strain E2348/69	[48]
MG1655	K-12 (F^−^ λ^−^)	[20]
EcM1	MG1655 Δ*fimA-H*	[21, 22]
EcM1∆*fliCD*	MG1655Δ*fimA-H* Δ*fliCD*	[49]
SIEC	EcM1-∆*yeeJ*::Ptac-eLEE2 ∆*yra*::Ptac-eLEE3 ∆*yfc*::Ptac-eEscD ∆*yebT*::Ptac-eLEE4 ∆*yfaL*::Ptac-eLEE1	[19]
SIECΔp1	SIEC ∆*yfaL*:: ΔPtac-eLEE1*	[19]
EcM1-eEtgA	EcM1 Δ*sfm*::Ptac-*etgA*	This study
SIEC-eEtgA	SIEC Δ*sfm*::Ptac-*etgA*	This study
SIECΔp1-eEtgA	SIECΔp1 Δ*sfm*::Ptac-*etgA*	This study
SIEC-eLEE5	SIEC Δ*flu*::Ptac-eLEE5	[19]
SIEC-eLEE5-eEtgA	SIEC-eLEE5 Δ*sfm*::Ptac-*etgA*	This study
SIEC-eLEE5Δ*escN*	SIEC-eLEE5 Δ*escN*	This study
Plasmids		
pACBSR	Cm^R^; p15A ori, P_BAD_ I-SceI λRed genes	[50]
pKD4	Amp^R^, oriRγ, Km^R^-FRT cassette	[46]
pKD46	Amp^R^, oriR101, P_BAD_ λRed genes	[46]
pGEsfmPtacGFP	Km^R^; R6K ori, *sfm* HRs, Ptac-*gfp*^TCD^	[19]
pGEsfmPtacEtgA	Km^R^; R6K ori, *sfm* HRs, Ptac-*etgA*	This study
pGEΔ*escN*	Km^R^; R6K ori, *escN* HRs	[42]
pT3s-Bla	Tc^R^, pBR-ori, Ptac-EspF20Bla	[39]
pBAD18	KmR, pBR-ori, PBAD	[43]
pBAD18EspF20Bla	KmR, pBAD18, PBAD-EspF20Bla	This study
pBAD18Bla	KmR, pBAD18, PBAD-Bla	This study

*HRs* homology regions

### Plasmid construction

Standard methods of DNA manipulation and cloning by digestion with restriction enzymes and ligation were applied to construct the different plasmids [41]. The DNA polymerase Herculase II Fusion (Agilent Technologies) with proof-reading activity was used for PCR amplification of the fragments for subsequent cloning. Colony PCR screenings were performed with NZYTaq II 2× Green Master Mix (NZYTech, Lda.). The DNA sequence of all the constructs was determined by Sanger sequencing (Macrogen). The *etgA* gene without its endogenous promoter was amplified with the primer pair F_NotI_etgA and R_SpeI_etgA (Table 2) using genomic DNA from the strain EPEC E2348/69 as a template [42]. The restriction recognition sequences for the enzymes NotI and SpeI were included in the oligonucleotides to clone the resulting 0.5-kb amplicon into pGE*sfm*PtacGFP, replacing the GFP gene. The new plasmid, named pGE*sfm*PtacEtgA, was used to integrate the *etgA* gene under the control of the *tac* promoter at the *sfm* locus of the different strains, as described below. EspF20-Bla and Bla regions were amplified from pT3s-Bla [39] with primers F_SacI_RBS_EspF20/R_SpeI_Bla and F_SacI_HindIII_bla/R_SpeI_Bla (Table 2), respectively. Amplified regions and the backbone plasmid (pBAD18) [43] were digested with SacI and SpeI restriction enzymes and ligated, generating the plasmids pBAD18EspF20-Bla and pBAD18Bla.

**Table 2:** Oligonucleotides used in this study

Name	Sequence (5′–3′)^a^	Purpose
F_NotI_etgA	GCTTTGCGGCCGCATATTGAGTAACAACGTAAAAATGAAAAAAATAATACTGAGTATCATTCTC	Cloning of the *etgA* gene
R_SpeI_etgA	GTGACACTAGTTTAATCGATAATTTGCTCATTATTCTTTAA	Cloning of the *etgA* gene
F_SacI_RBS_EspF20	AATTCGAGCTCAAGCTTAAGAAGGAGATATACATATGCTTAATGGAATTAGTAACGCTGCTTC	Cloning of EspF20-bla
F_SacI_HindIII_bla	ATGCTGAGCTCAAGCTTAAGAAGGAGATATACATATGGGAAGCCACCCAGAAACGCTGGTGAAAGTAAAAG	Cloning of Bla
R_SpeI_Bla	CATGCACTAGTTTACCAATGCTTAATCAGTGAGGCACC	Cloning of EspF20-bla and Bla
Int_UP_sfm	TTCTTCACAAGCCTTGCGTTTGCTTGCGAC	Check *etgA* integration
F.check.DescN	CGGCGTACAAGAAACGCGTTATTTG	Check *escN* deletion
R.check.DescN	CGAAAACATAGTCTTTTTTATG	Check *escN* deletion
tolC-kmUP	ATGAAGAAATTGCTCCCCATTCTTATCGGCCTGAGCCTTTCTGGGTTCAG*gtgtaggctggagctgcttc*	Deletion of *tolC*
tolC-KmDWN	TCAGTTACGGAAAGGGTTATGACCGTTACTGGTGGTAGTGCGTGCGGATG*catatgaatatcctccttagt*	Deletion of *tolC*
TolC-UP-1	CGGGAATTCTCTAGACTGCTTCACCACAAGGAATGCAAATG	Check *tolC::km1*
TolC-DW-1	CCGCGGATCCAAGCTTGGCCGAAGCCCCGTCGTCGTCATCA	Check *tolC::km1*
k2	CGGTGCCCTGAATGAACTGC	Check *tolC::km1*

^a^Restriction sites used for cloning are underlined

### Genome modifications

The chromosomal integration of the *etgA* gene in the strains EcM1, SIEC, SIECΔp1 and SIEC-eLEE5 was carried out by following a scarless genome editing strategy based on two sequential homologous recombination events, the second promoted by the expression of the restriction enzyme I-SceI and the λRed proteins from the plasmid pACBSR (Table 1), as described [19, 44]. The corresponding strain carrying pACBSR was electroporated with the pir-dependent suicide plasmid pGE*sfm*PtacEtgA (Table 1) for its integration. The resulting Km^R^ Cm^R^ colonies were grown overnight (O/N) at 37 °C with agitation (250 rpm) in LB with Km and Cm. The next day, the O/N cultures were diluted 1:100 in fresh LB with only Cm, and after reaching OD_600nm_ 0.4–0.5, the expression of I-SceI and the λRed proteins was induced with 0.4% (w/v) ARA for 5 h. The induced cultures were plated on LB agar with Cm. The Cm^R^ colonies were streaked onto LB agar with and without Km to confirm the loss of the vector sequences. The integration of the *etgA* gene at the *sfm* locus was assessed by colony PCR with the primer pair Int_UP_sfm/R_SpeI_etgA (Table 1). The plasmid pACBSR was eliminated from the final strains by passaging the cultures in LB without antibiotics. The resulting strains were named EcM1-eEtgA, SIEC-eEtgA, SIECΔp1-eEtgA and SIEC-eLEE5-eEtgA.

The control strain SIEC-eLEE5Δ*escN* was created following the same strategy as above to delete the cognate gene *escN* of SIEC-eLEE5. In this case, the suicide plasmid pGEΔ*escN* was used for deletion [42] (Table 2), which was confirmed by colony PCR using the primer pair F.check.ΔescN/R.check.ΔescN (Table 2). The *E. coli* UT5600 Δ*tolC::km1* strain was generated by deletion of the *tolC* gene from the strain UT5600 [45] using the one-step inactivation method with PCR products [46]. Briefly, a PCR fragment containing a Km^R^ gene cassette with flanking *FRT* sites and 50 bp of the 5′ and 3′ *tolC* ORF was obtained by amplification with oligonucleotides tolC-KmUP and tolC-KmDWN (Table 2) using plasmid pKD4 as template [46] (Table 1). The amplified DNA product was digested with DpnI (to eliminate pKD4), gel-purified, and electroporated into *E. coli* UT5600 carrying pKD46 [46] (Table 1), which was grown and induced with ARA (0.4% w/v) before electroporation. The Km^R^ colonies grown after electroporation were isolated and Δ*tolC::km1* mutants were confirmed by PCR of chromosomal DNA with primers TolC-UP-1 (upstream of tolC ORF) and TolC-DW-1 (downstream of tolC ORF) and k2 (internal for Km^R^) and TolC-DW-1 (Table 2).

### Analysis of culture supernatants by SDS-PAGE and quantification of secreted proteins

To induce the T3SS components in the different strains, O/N cultures were diluted 1:100 in capped Falcon tubes with 5 ml of LB containing 0.1 mM IPTG, and incubated at 37 °C with agitation (160 rpm) for 6 h. Two milliliters of each induced culture was centrifuged at 6000×*g* for 5 min and 1.8 ml of supernatant was transferred to a new microcentrifuge tube containing 0.2 ml of 100% w/v trichloroacetic acid (TCA; Merck) for protein precipitation. After thorough mixing, the tubes were incubated for 60 min on ice, and subsequently centrifugated at 20,000×*g* for 15 min at 4 °C. The resulting protein pellets were rinsed with cold acetone (− 20 °C), air-dried, and resuspended in 30 µl of SDS-PAGE sample buffer [60 mM Tris–HCl pH 6.8, 1% (w/v) SDS, 5% (v/v) glycerol, 0.005% (w/v) bromophenol blue and 1% (v/v) 2-mercaptoethanol]. Samples were boiled for 10 min and loaded into 15% polyacrylamide SDS gels [47]. Electrophoresis was done following standard methods on the Miniprotean III system (Bio-Rad). Gels were stained with Coomassie Blue R-250 (Bio-Rad) and images were acquired with a Gel Doc XR + System (Bio-Rad). Image Lab Software (Bio-Rad) was used for relative quantification of EspB and EspA proteins, taking as a reference (100% of secretion) the corresponding protein bands in SIEC. The supernatants from nine induced cultures per strain were analyzed.

### Hemolysis assay

The hemolytic capacity of the *E. coli* strains was determined as described [19] using New Zealand White rabbit erythrocytes (Gabinete Veterinario, Universidad Autónoma de Madrid). Blood was treated with a final concentration of 0.1% (w/v) EDTA pH 7.5 to prevent coagulation. Erythrocytes from 5 ml of blood were collected by centrifugation (3500×*g*, 15 min, room temperature [RT]), and washed three times with one volume of 0.9% NaCl with slow centrifugation (1000×*g*, 10 min, RT) between washes. The erythrocyte suspension was diluted to 4% in DMEM, and 0.5 ml of this suspension was mixed with 0.5 ml of induced bacterial culture (OD_600_ 0.4). The mix was centrifuged (2500×*g*, 1 min, RT) to induce contact of the bacteria with the erythrocytes and then incubated at 37 °C with 5% CO_2_ during 4 h. The pellet was then gently resuspended and centrifuged (12,000×*g*, 1 min, RT), and hemoglobin released into the supernatant was measured at 450 nm (OD_450_) in a spectrophotometer (Ultraspec 3100 pro, Amersham Biosciences). The background average OD_450_ value obtained with the control strain EcM1 was subtracted from the values of all other samples, and the value of SIEC was taken as reference (100%).

### Swimming motility assay

The motility of *E. coli* strains was evaluated as follows: soft-agar petri dishes (150 mm) were prepared with LB containing 0.3% (w/v) Bacto-agar (Difco) and 0.1 mM IPTG. Fresh bacterial colonies of the indicated strains were spiked onto the agar in a regular distribution. The plates were incubated for 8 h at 37 °C and at RT O/N. The diameter of the colonies was then measured using the average value of EcM1 as 100% motility.

### Infection of cell cultures and fluorescence confocal microscopy

The human cell line HeLa (CCL-2, ATCC) was infected with different SIEC-eLEE5-derived strains for actin pedestal observation. HeLa cells were seeded in 24-well tissue plates (10^5^ cells/well) with sterile coverslips at the bottom and incubated O/N with DMEM supplemented with 10% fetal bovine serum (FBS; Sigma) and 2 mM l-glutamine, at 37 °C with 5% CO_2_ under static conditions. In parallel, single colonies of the SIEC-eLEE5 strains were also grown O/N in LB at 37 °C and 160 rpm. The next day, SIEC-eLEE5 cultures were diluted 1:100 in fresh LB with 0.1 mM IPTG and induced for 2.5 h at 37 °C and 160 rpm. After induction, bacterial cultures were diluted to OD_600_ 0.05 in serum-free DMEM containing IPTG. Prior to the addition of bacteria, the cell cultures were washed three times with serum-free DMEM to completely eliminate FBS from the culture medium. DMEM-diluted bacteria were added over the cells (0.5 ml/well) and the plate was incubated at 37 °C with 5% CO_2_ under static conditions. After 90 min, wells were washed three times with serum-free DMEM with IPTG to remove the excess bacteria, and the plate was incubated for a further 90 min. Infections were stopped by three washes with sterile PBS, and samples were fixed with 4% (w/v) paraformaldehyde (in PBS, 20 min, RT). Samples were then washed with PBS three times and permeabilized by incubation with 0.1% (v/v) saponin (Sigma) in PBS for 10 min. To stain the SIEC-eLEE5 strains, bacteria were incubated with a polyclonal rabbit anti-intimin280 antibody (1:500) in PBS with 10% (v/v) goat serum (Sigma) and incubated for 60 min at RT. Coverslips were washed three times with PBS and were then incubated for 45 min with a goat anti-rabbit secondary antibody conjugated to Alexa488 (1:500; Life Technologies) in PBS with 10% goat serum together with phalloidin-tetramethylrhodamine (TRITC) (1:500; Sigma) and 4′,6-diamidino-2-phenylindole (DAPI) (1:500; Sigma) to label F-actin and DNA, respectively. Coverslips were washed three times with PBS after incubation and 4 µl of ProLong Gold anti-fade reagent (Life Technologies) was added before mounting on microscope slides. Cells were observed with an SP5 confocal microscope (Leica) using the 100× objective and an additional 2.5-fold magnification. Images were processed using ImageJ software (NIH).

### Protein translocation assay

Protein translocation quantification was assessed using the LiveBLAzer™ FRET B/G Loading Kit with CCF2-AM (ThermoFisher Scientific). Briefly, HeLa cells were seeded 2 days prior to the experiment in a tissue culture-treated 96-well black plate with a flat clear bottom (Corning) at 10^4^ cells per well. A minimum of three wells per experimental condition, including non-infected cells, were prepared. Wells with no cells were also used as blanks. The plate was incubated at 37 °C with 5% CO_2_ under static conditions until the day of the experiment (48 h). The day before the experiment, bacteria with pBAD18EspF20-Bla were grown O/N from a single colony in 5 ml of LB with Km at 160 rpm and 37 °C. The next day, bacterial cultures were diluted 1:100 in 5 ml of LB containing IPTG at 0.1 mM. Bacteria were grown with 0.1 mM IPTG for 2 h with agitation (160 rpm) at 37 °C, before 0.4% (w/v) ARA was added. After one more hour of induction with agitation (160 rpm) at 37 °C, bacteria were diluted to 0.1 OD_600_/ml in serum-free DMEM containing IPTG and ARA. Prior to infection, cell cultures were washed three times with serum-free DMEM to completely eliminate FBS from the culture medium. DMEM-diluted bacteria were added over the cells (200 µl/well) and the plate was incubated for 90 min at 37 °C with 5% CO_2_ under static conditions. Subsequently, the wells were washed three times with preheated phenol red-free HBSS (Gibco). Then, each well, including blank wells, was covered with the β-lactamase substrate mix (100 µl of HBSS + 20 µl of 6× CCF2/AM solution freshly prepared with the CCF2/AM loading kit; CCF2/AM final concentration of 1 µM). The plate was incubated at RT in the dark for 90 min before being loaded into a SpectraMax iD5 Multi-mode Microplate Reader (Molecular Devices) for double fluorescence determination: excitation at 409 nm wavelength, emission at 450 nm (blue fluorescence) and 520 nm (green fluorescence). Emission values of blank wells at the corresponding wavelengths were subtracted from each well. The relative translocation levels were obtained by calculating the fluorescence emission ratio between 450 and 520 nm (blue emission/green emission) for every well. Each experimental condition was normalized to the emission ratio of the non-infected cells.

## Data Availability

All the data generated in the study are included in the present manuscript. All the materials described are available from the corresponding author upon reasonable request.
